# Defense Mechanisms and Ego Adaptation in Coping With Chronic Illness

**DOI:** 10.1192/j.eurpsy.2026.11058

**Published:** 2026-07-31

**Authors:** E. M. Jetter, D. Valle, H. Nyugen, B. R. Carr

**Affiliations:** 1 College of Medicine; 2 Department of Psychiatry, University of Florida, Gainesville, United States

## Abstract

**Introduction:**

Chronic illness evokes defenses that help patients endure yet complicate care. Freud first described them as unconscious strategies to ward off distress (Freud A. Writings of Anna Freud 1936; 2:3-191). Vaillant later organized them into adaptive and maladaptive hierarchies (Vaillant GE. Ego Mechanisms of Defense 1992). Together these frameworks show how defenses both protect and hinder adaptation, explaining why some patients thrive while others falter in coping.

**Objectives:**

To examine how ego psychology clarifies the role of defense mechanisms in chronic illness and to outline applications for medical education.

**Methods:**

A narrative review was conducted of psychoanalytic literature on ego defenses, alongside psychosomatic and behavioral health research. Clinical descriptions of coping in chronic illness were analyzed for defensive patterns and their effects. Educational models for teaching defenses in communication and professional identity were also reviewed.

**Results:**

In chronic illness, defenses influence adherence, trust, and the clinician’s experience of care (Table 1). Adaptive defenses foster resilience and therapeutic alliance; small-group role-play and case discussions help trainees recognize and reinforce them. Intermediate defenses engage cognition but limit emotion, leading to mixed adherence and clinician frustration; feedback-based workshops with simulated patients train learners to balance reasoning with empathy. Maladaptive defenses shield distress but risk nonadherence and conflict; reflection groups guide learners to recognize these defenses, monitor their reactions, and maintain therapeutic boundaries.

**Image 1:**
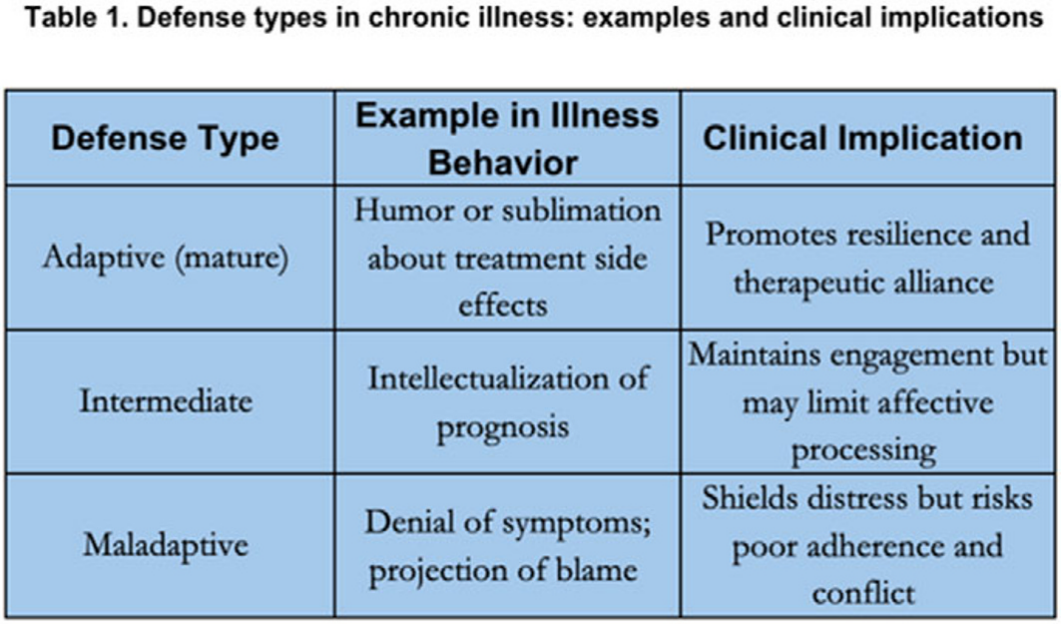
Image 1: Long description.

**Conclusions:**

Viewing coping through ego psychology reframes defenses as dynamic strategies rather than obstacles. For clinicians, this perspective highlights patient behavior as meaningful, not oppositional. For educators, it offers tools to teach recognition of defenses, preserve trust, and sustain therapeutic momentum. Integrating defenses within a psychoanalytic framework prepares physicians for the psychological as well as biomedical realities of chronic illness.

**Disclosure of Interest:**

None Declared

## Long descriptions

### Image 1: Long description

The table consists of three columns and three data rows.

Column headers from left to right are Defense Type, Example in Illness Behavior, and Clinical Implication.

Row 1:

- Defense Type: Adaptive (mature).

- Example in Illness Behavior: Humor or sublimation about treatment side effects.

- Clinical Implication: Promotes resilience and therapeutic alliance.

Row 2:

- Defense Type: Intermediate.

- Example in Illness Behavior: Intellectualization of prognosis.

- Clinical Implication: Maintains engagement but may limit affective processing.

Row 3:

- Defense Type: Maladaptive.

- Example in Illness Behavior: Denial of symptoms; projection of blame.

- Clinical Implication: Shields distress but risks poor adherence and conflict.

Navigate back to Image 1:.

